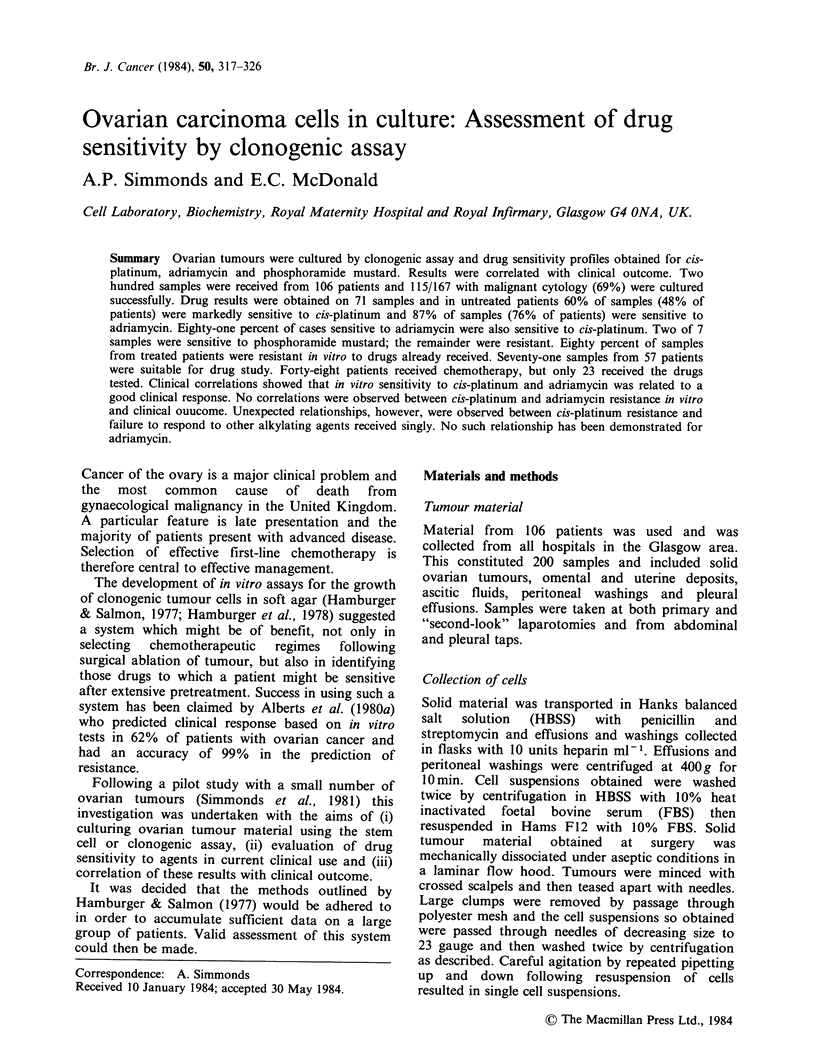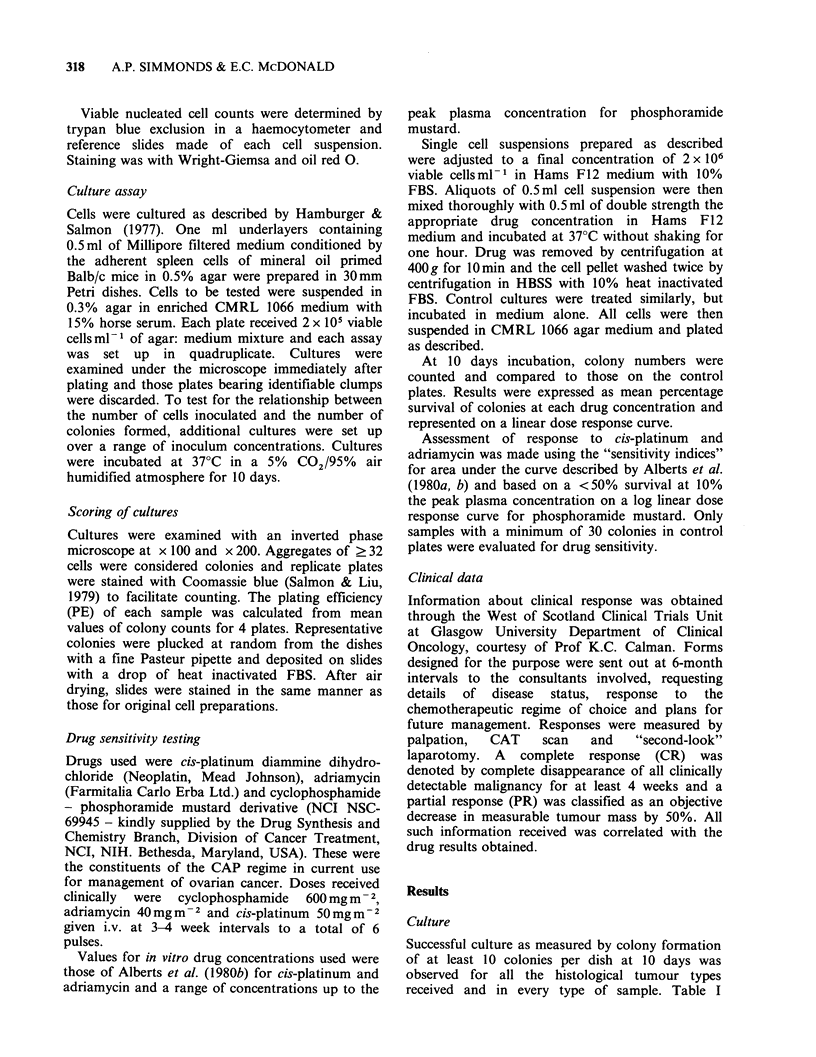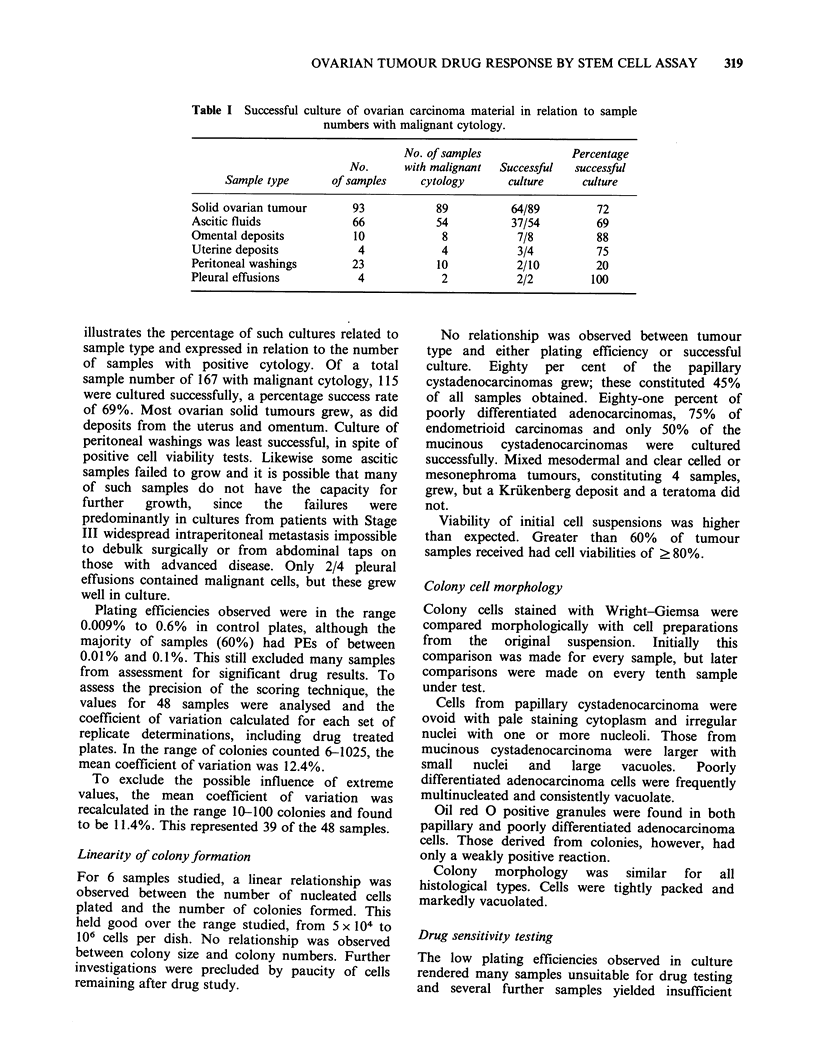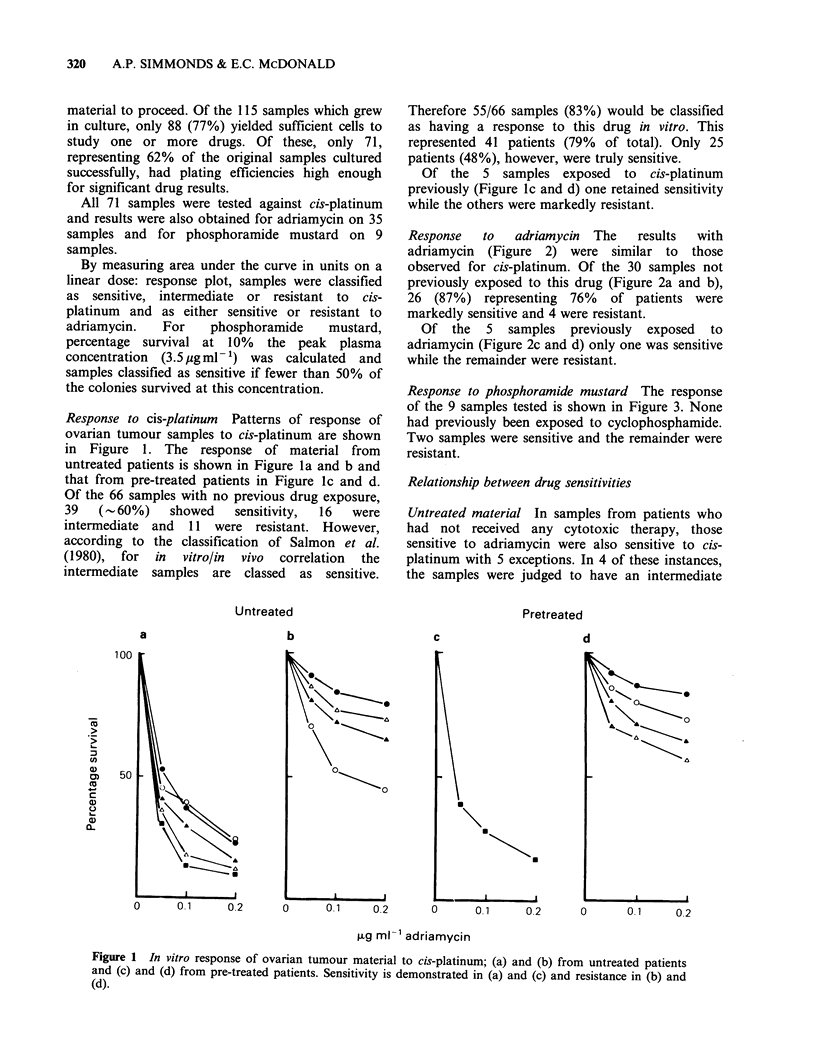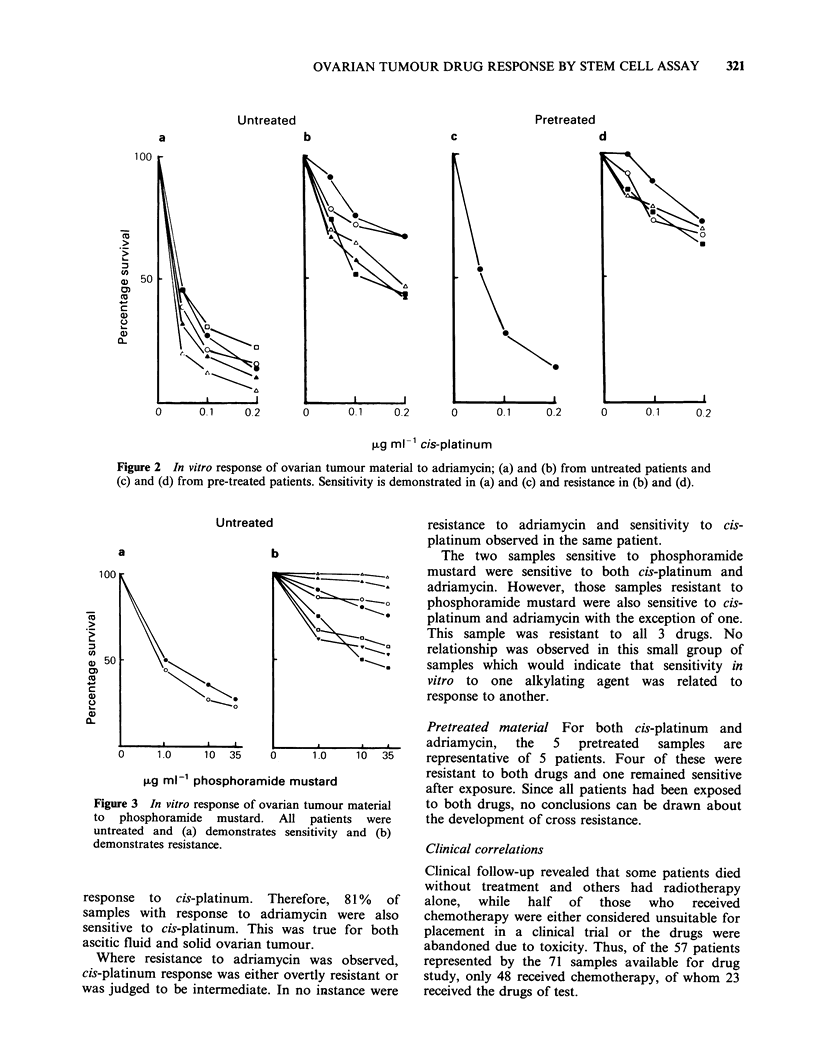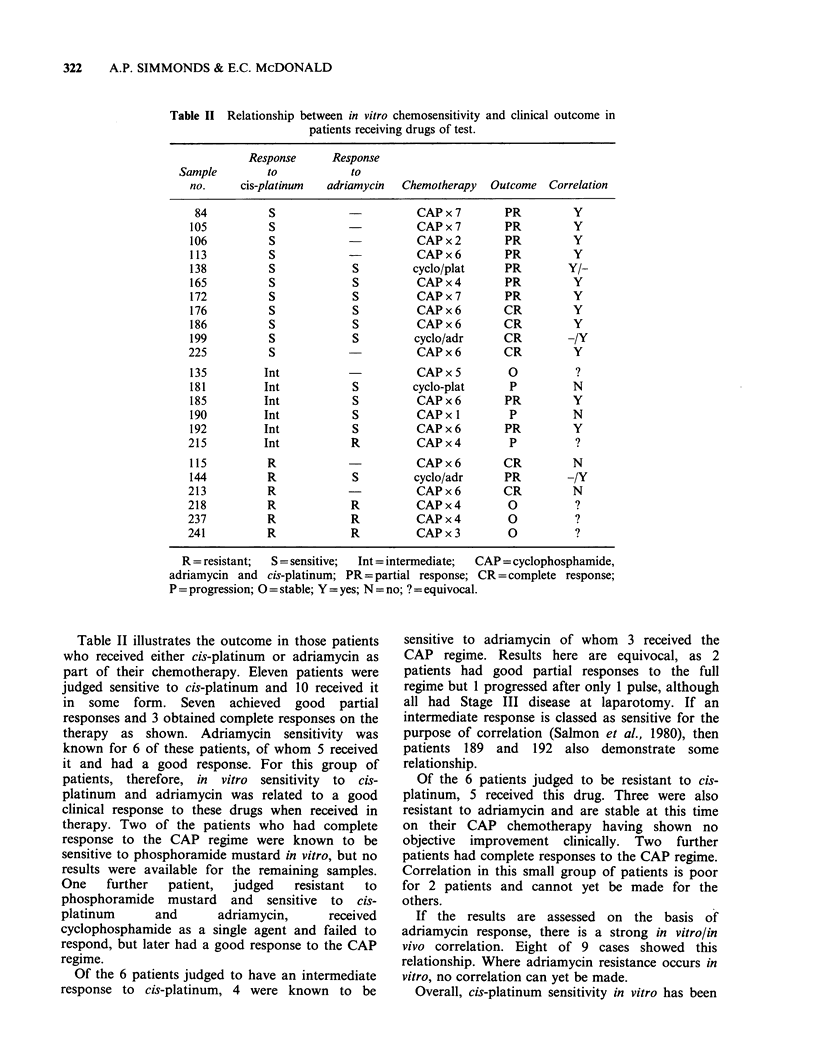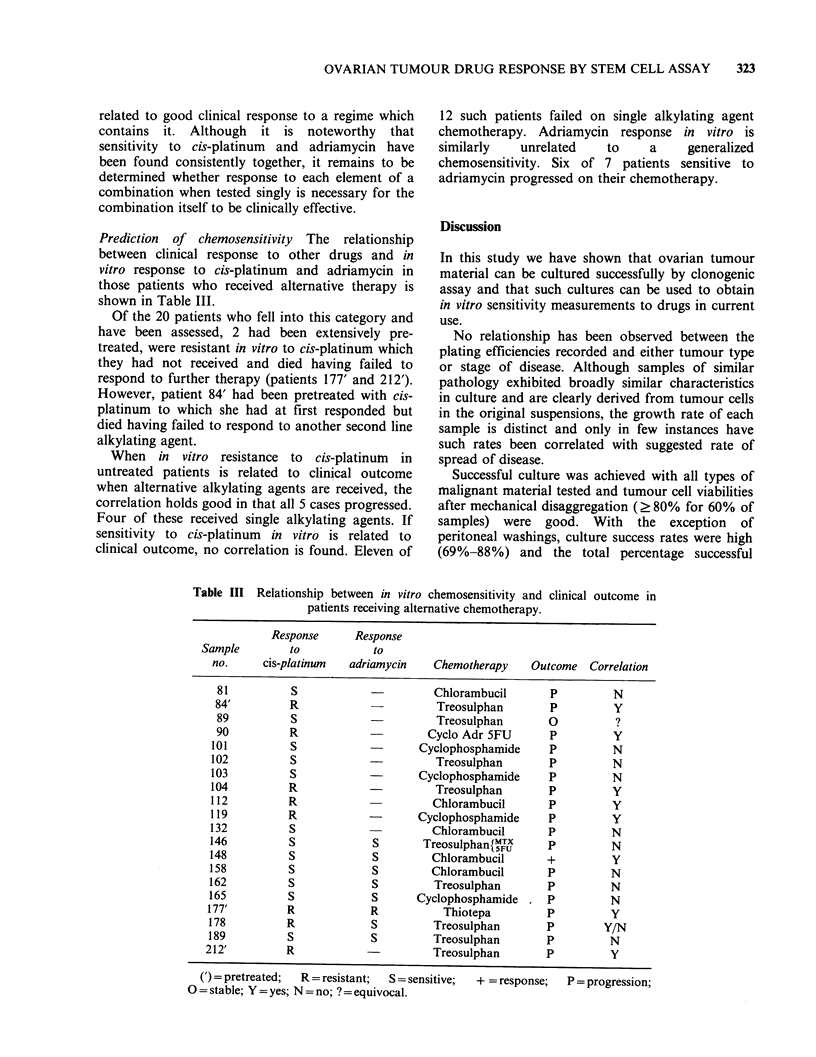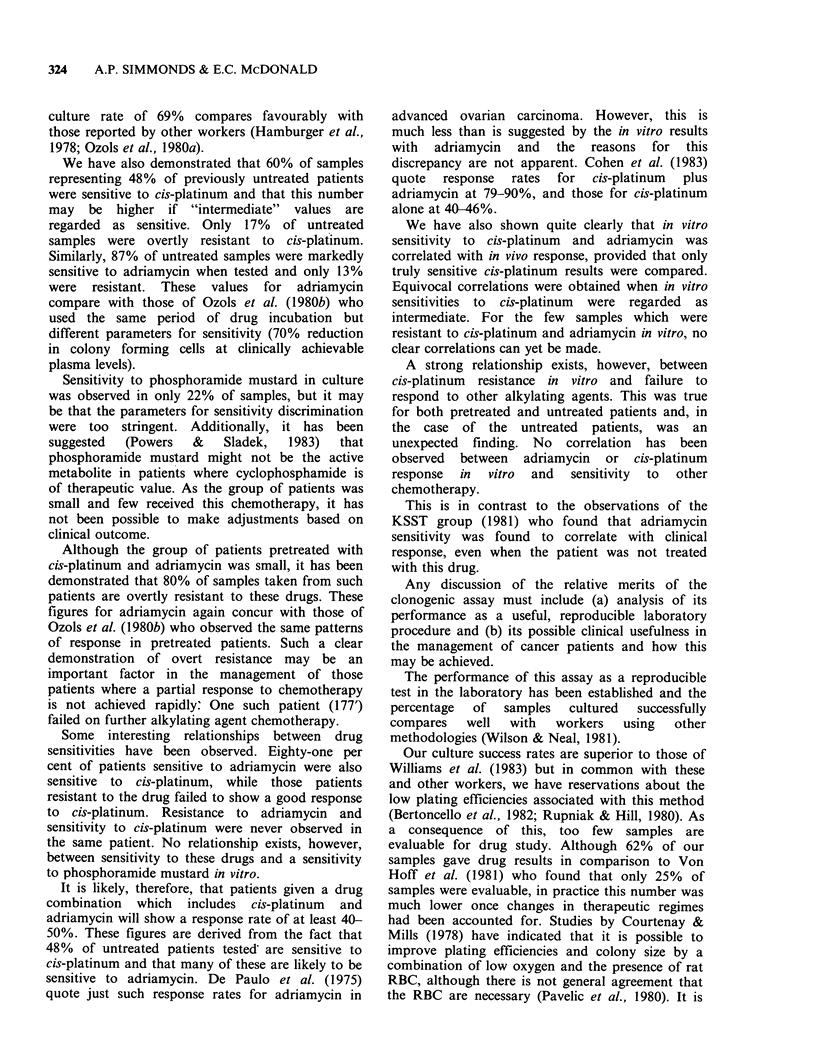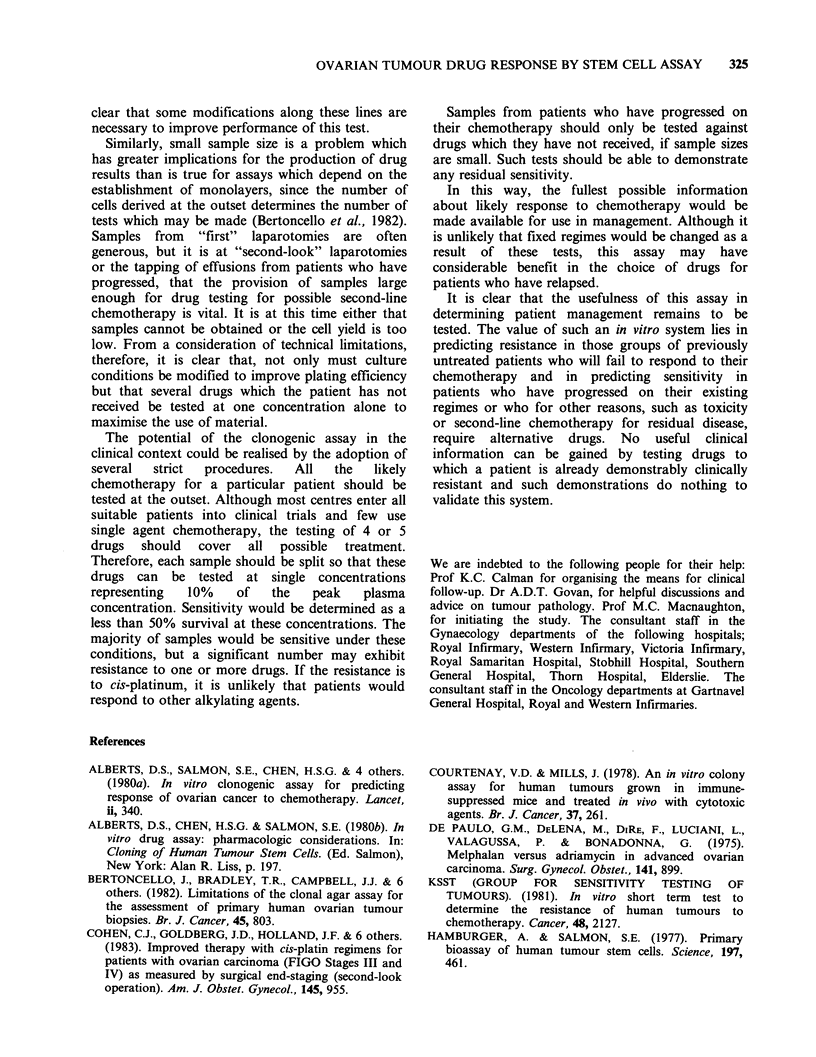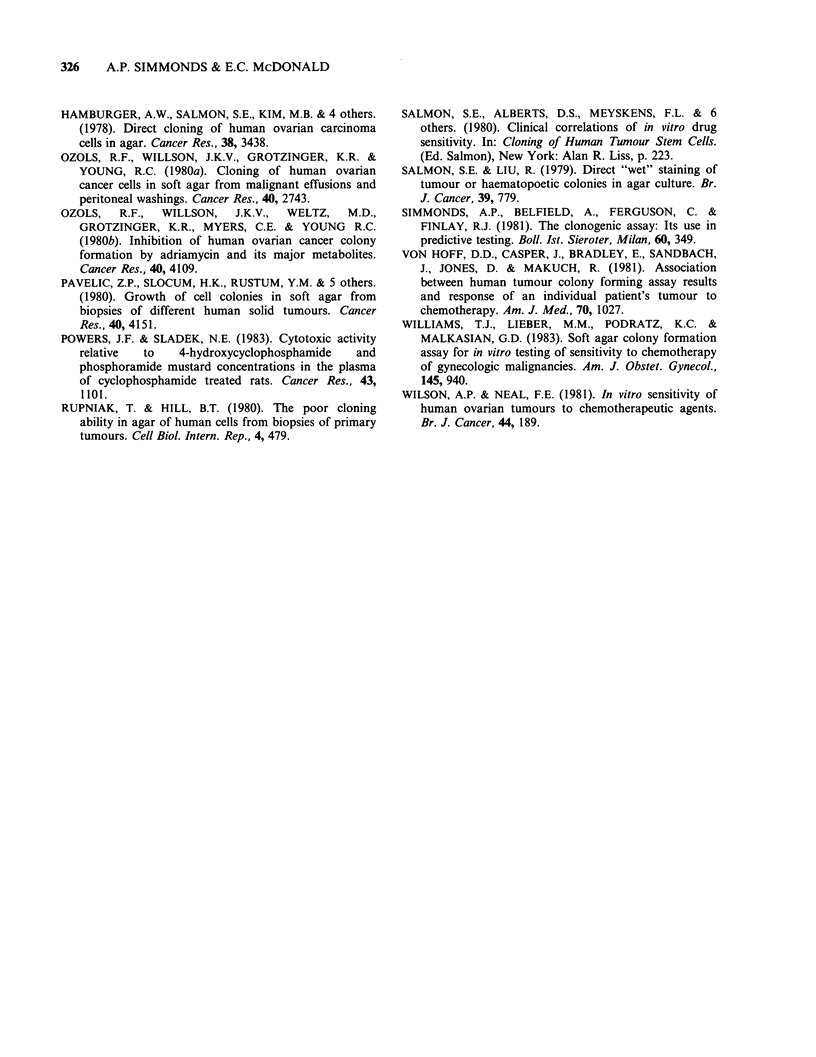# Ovarian carcinoma cells in culture: assessment of drug sensitivity by clonogenic assay.

**DOI:** 10.1038/bjc.1984.178

**Published:** 1984-09

**Authors:** A. P. Simmonds, E. C. McDonald

## Abstract

Ovarian tumours were cultured by clonogenic assay and drug sensitivity profiles obtained for cis-platinum, adriamycin and phosphoramide mustard. Results were correlated with clinical outcome. Two hundred samples were received from 106 patients and 115/167 with malignant cytology (69%) were cultured successfully. Drug results were obtained on 71 samples and in untreated patients 60% of samples (48% of patients) were markedly sensitive to cis-platinum and 87% of samples (76% of patients) were sensitive to adriamycin. Eighty-one percent of cases sensitive to adriamycin were also sensitive to cis-platinum. Two of 7 samples were sensitive to phosphoramide mustard; the remainder were resistant. Eighty percent of samples from treated patients were resistant in vitro to drugs already received. Seventy-one samples from 57 patients were suitable for drug study. Forty-eight patients received chemotherapy, but only 23 received the drugs tested. Clinical correlations showed that in vitro sensitivity to cis-platinum and adriamycin was related to a good clinical response. No correlations were observed between cis-platinum and adriamycin resistance in vitro and clinical outcome. Unexpected relationships, however, were observed between cis-platinum resistance and failure to respond to other alkylating agents received singly. No such relationship has been demonstrated for adriamycin.


Br. J. Cancer (1984), **50**, 317–326

### Ovarian carcinoma cells in culture: Assessment of drug sensitivity by clonogenic assay

A.P. Simmonds and E.C. McDonald

*Cell Laboratory, Biochemistry, Royal Maternity Hospital and Royal Infirmary, Glasgow G4 0NA, UK.*

**Summary** Ovarian tumours were cultured by clonogenic assay and drug sensitivity profiles obtained for *cis*-platinum, adriamycin and phosphoramide mustard. Results were correlated with clinical outcome. Two hundred samples were received from 106 patients and 115/167 with malignant cytology (69%) were cultured successfully. Drug results were obtained on 71 samples and in untreated patients 60% of samples (48% of patients) were markedly sensitive to *cis*-platinum and 87% of samples (76% of patients) were sensitive to adriamycin. Eighty-one percent of cases sensitive to adriamycin were also sensitive to *cis*-platinum. Two of 7 samples were sensitive to phosphoramide mustard; the remainder were resistant. Eighty percent of samples from treated patients were resistant *in vitro* to drugs already received. Seventy-one samples from 57 patients were suitable for drug study. Forty-eight patients received chemotherapy, but only 23 received the drugs tested. Clinical correlations showed that *in vitro* sensitivity to *cis*-platinum and adriamycin was related to a good clinical response. No correlations were observed between *cis*-platinum and adriamycin resistance *in vitro* and clinical ouucome. Unexpected relationships, however, were observed between *cis*-platinum resistance and failure to respond to other alkylating agents received singly. No such relationship has been demonstrated for adriamycin.

Cancer of the ovary is a major clinical problem and the most common cause of death from gynaecological malignancy in the United Kingdom. A particular feature is late presentation and the majority of patients present with advanced disease. Selection of effective first-line chemotherapy is therefore central to effective management.

The development of *in vitro* assays for the growth of clonogenic tumour cells in soft agar (Hamburger & Salmon, 1977; Hamburger *et al.*, 1978) suggested a system which might be of benefit, not only in selecting chemotherapeutic regimes following surgical ablation of tumour, but also in identifying those drugs to which a patient might be sensitive after extensive pretreatment. Success in using such a system has been claimed by Alberts *et al.* (1980*a*) who predicted clinical response based on *in vitro* tests in 62% of patients with ovarian cancer and had an accuracy of 99% in the prediction of resistance.

Following a pilot study with a small number of ovarian tumours (Simmonds *et al.*, 1981) this investigation was undertaken with the aims of (i) culturing ovarian tumour material using the stem cell or clonogenic assay, (ii) evaluation of drug sensitivity to agents in current clinical use and (iii) correlation of these results with clinical outcome.

It was decided that the methods outlined by Hamburger & Salmon (1977) would be adhered to in order to accumulate sufficient data on a large group of patients. Valid assessment of this system could then be made.

Correspondence: A. Simmonds
Received 10 January 1984; accepted 30 May 1984.

### Materials and methods

### Tumour material

Material from 106 patients was used and was collected from all hospitals in the Glasgow area. This constituted 200 samples and included solid ovarian tumours, omental and uterine deposits, ascitic fluids, peritoneal washings and pleural effusions. Samples were taken at both primary and "second-look" laparotomies and from abdominal and pleural taps.

### Collection of cells

Solid material was transported in Hanks balanced salt solution (HBSS) with penicillin and streptomycin and effusions and washings collected in flasks with 10 units heparin $ml^{-1}$. Effusions and peritoneal washings were centrifuged at 400 *g* for 10 min. Cell suspensions obtained were washed twice by centrifugation in HBSS with 10% heat inactivated foetal bovine serum (FBS) then resuspended in Hams F12 with 10% FBS. Solid tumour material obtained at surgery was mechanically dissociated under aseptic conditions in a laminar flow hood. Tumours were minced with crossed scalpels and then teased apart with needles. Large clumps were removed by passage through polyester mesh and the cell suspensions so obtained were passed through needles of decreasing size to 23 gauge and then washed twice by centrifugation as described. Careful agitation by repeated pipetting up and down following resuspension of cells resulted in single cell suspensions.

© The Macmillan Press Ltd., 1984

Viable nucleated cell counts were determined by trypan blue exclusion in a haemocytometer and reference slides made of each cell suspension. Staining was with Wright-Giemsa and oil red O.

*Culture assay*

Cells were cultured as described by Hamburger & Salmon (1977). One ml underlayers containing 0.5 ml of Millipore filtered medium conditioned by the adherent spleen cells of mineral oil primed Balb/c mice in 0.5% agar were prepared in 30 mm Petri dishes. Cells to be tested were suspended in 0.3% agar in enriched CMRL 1066 medium with 15% horse serum. Each plate received $2\times10^5$ viable cells $ml^{-1}$ of agar: medium mixture and each assay was set up in quadruplicate. Cultures were examined under the microscope immediately after plating and those plates bearing identifiable clumps were discarded. To test for the relationship between the number of cells inoculated and the number of colonies formed, additional cultures were set up over a range of inoculum concentrations. Cultures were incubated at 37°C in a 5% $CO_2$/95% air humidified atmosphere for 10 days.

*Scoring of cultures*

Cultures were examined with an inverted phase microscope at ×100 and ×200. Aggregates of ≥32 cells were considered colonies and replicate plates were stained with Coomassie blue (Salmon & Liu, 1979) to facilitate counting. The plating efficiency (PE) of each sample was calculated from mean values of colony counts for 4 plates. Representative colonies were plucked at random from the dishes with a fine Pasteur pipette and deposited on slides with a drop of heat inactivated FBS. After air drying, slides were stained in the same manner as those for original cell preparations.

*Drug sensitivity testing*

Drugs used were *cis*-platinum diammine dihydrochloride (Neoplatin, Mead Johnson), adriamycin (Farmitalia Carlo Erba Ltd.) and cyclophosphamide – phosphoramide mustard derivative (NCI NSC-69945 – kindly supplied by the Drug Synthesis and Chemistry Branch, Division of Cancer Treatment, NCI, NIH. Bethesda, Maryland, USA). These were the constituents of the CAP regime in current use for management of ovarian cancer. Doses received clinically were cyclophosphamide 600 mg $m^{-2}$, adriamycin 40 mg $m^{-2}$ and *cis*-platinum 50 mg $m^{-2}$ given i.v. at 3–4 week intervals to a total of 6 pulses.

Values for *in vitro* drug concentrations used were those of Alberts *et al.* (1980*b*) for *cis*-platinum and adriamycin and a range of concentrations up to the peak plasma concentration for phosphoramide mustard.

Single cell suspensions prepared as described were adjusted to a final concentration of $2\times10^6$ viable cells $ml^{-1}$ in Hams F12 medium with 10% FBS. Aliquots of 0.5 ml cell suspension were then mixed thoroughly with 0.5 ml of double strength the appropriate drug concentration in Hams F12 medium and incubated at 37°C without shaking for one hour. Drug was removed by centrifugation at 400 *g* for 10 min and the cell pellet washed twice by centrifugation in HBSS with 10% heat inactivated FBS. Control cultures were treated similarly, but incubated in medium alone. All cells were then suspended in CMRL 1066 agar medium and plated as described.

At 10 days incubation, colony numbers were counted and compared to those on the control plates. Results were expressed as mean percentage survival of colonies at each drug concentration and represented on a linear dose response curve.

Assessment of response to *cis*-platinum and adriamycin was made using the "sensitivity indices" for area under the curve described by Alberts *et al.* (1980*a, b*) and based on a <50% survival at 10% the peak plasma concentration on a log linear dose response curve for phosphoramide mustard. Only samples with a minimum of 30 colonies in control plates were evaluated for drug sensitivity.

*Clinical data*

Information about clinical response was obtained through the West of Scotland Clinical Trials Unit at Glasgow University Department of Clinical Oncology, courtesy of Prof K.C. Calman. Forms designed for the purpose were sent out at 6-month intervals to the consultants involved, requesting details of disease status, response to the chemotherapeutic regime of choice and plans for future management. Responses were measured by palpation, CAT scan and "second-look" laparotomy. A complete response (CR) was denoted by complete disappearance of all clinically detectable malignancy for at least 4 weeks and a partial response (PR) was classified as an objective decrease in measurable tumour mass by 50%. All such information received was correlated with the drug results obtained.

### Results

*Culture*

Successful culture as measured by colony formation of at least 10 colonies per dish at 10 days was observed for all the histological tumour types received and in every type of sample. Table I

**Table I** Successful culture of ovarian carcinoma material in relation to sample numbers with malignant cytology.

| *Sample type* | *No. of samples* | *No. of samples with malignant cytology* | *Successful culture* | *Percentage successful culture* |
|---|---|---|---|---|
| Solid ovarian tumour | 93 | 89 | 64/89 | 72 |
| Ascitic fluids | 66 | 54 | 37/54 | 69 |
| Omental deposits | 10 | 8 | 7/8 | 88 |
| Uterine deposits | 4 | 4 | 3/4 | 75 |
| Peritoneal washings | 23 | 10 | 2/10 | 20 |
| Pleural effusions | 4 | 2 | 2/2 | 100 |

illustrates the percentage of such cultures related to sample type and expressed in relation to the number of samples with positive cytology. Of a total sample number of 167 with malignant cytology, 115 were cultured successfully, a percentage success rate of 69%. Most ovarian solid tumours grew, as did deposits from the uterus and omentum. Culture of peritoneal washings was least successful, in spite of positive cell viability tests. Likewise some ascitic samples failed to grow and it is possible that many of such samples do not have the capacity for further growth, since the failures were predominantly in cultures from patients with Stage III widespread intraperitoneal metastasis impossible to debulk surgically or from abdominal taps on those with advanced disease. Only 2/4 pleural effusions contained malignant cells, but these grew well in culture.

Plating efficiencies observed were in the range 0.009% to 0.6% in control plates, although the majority of samples (60%) had PEs of between 0.01% and 0.1%. This still excluded many samples from assessment for significant drug results. To assess the precision of the scoring technique, the values for 48 samples were analysed and the coefficient of variation calculated for each set of replicate determinations, including drug treated plates. In the range of colonies counted 6–1025, the mean coefficient of variation was 12.4%.

To exclude the possible influence of extreme values, the mean coefficient of variation was recalculated in the range 10–100 colonies and found to be 11.4%. This represented 39 of the 48 samples.

*Linearity of colony formation*

For 6 samples studied, a linear relationship was observed between the number of nucleated cells plated and the number of colonies formed. This held good over the range studied, from $5 \times 10^4$ to $10^6$ cells per dish. No relationship was observed between colony size and colony numbers. Further investigations were precluded by paucity of cells remaining after drug study.

No relationship was observed between tumour type and either plating efficiency or successful culture. Eighty per cent of the papillary cystadenocarcinomas grew; these constituted 45% of all samples obtained. Eighty-one percent of poorly differentiated adenocarcinomas, 75% of endometrioid carcinomas and only 50% of the mucinous cystadenocarcinomas were cultured successfully. Mixed mesodermal and clear celled or mesonephroma tumours, constituting 4 samples, grew, but a Krükenberg deposit and a teratoma did not.

Viability of initial cell suspensions was higher than expected. Greater than 60% of tumour samples received had cell viabilities of $\geq 80\%$.

*Colony cell morphology*

Colony cells stained with Wright–Giemsa were compared morphologically with cell preparations from the original suspension. Initially this comparison was made for every sample, but later comparisons were made on every tenth sample under test.

Cells from papillary cystadenocarcinoma were ovoid with pale staining cytoplasm and irregular nuclei with one or more nucleoli. Those from mucinous cystadenocarcinoma were larger with small nuclei and large vacuoles. Poorly differentiated adenocarcinoma cells were frequently multinucleated and consistently vacuolate.

Oil red O positive granules were found in both papillary and poorly differentiated adenocarcinoma cells. Those derived from colonies, however, had only a weakly positive reaction.

Colony morphology was similar for all histological types. Cells were tightly packed and markedly vacuolated.

*Drug sensitivity testing*

The low plating efficiencies observed in culture rendered many samples unsuitable for drug testing and several further samples yielded insufficient material to proceed. Of the 115 samples which grew in culture, only 88 (77%) yielded sufficient cells to study one or more drugs. Of these, only 71, representing 62% of the original samples cultured successfully, had plating efficiencies high enough for significant drug results.

All 71 samples were tested against *cis*-platinum and results were also obtained for adriamycin on 35 samples and for phosphoramide mustard on 9 samples.

By measuring area under the curve in units on a linear dose: response plot, samples were classified as sensitive, intermediate or resistant to *cis*-platinum and as either sensitive or resistant to adriamycin. For phosphoramide mustard, percentage survival at 10% the peak plasma concentration (3.5 $\mu g ml^{-1}$) was calculated and samples classified as sensitive if fewer than 50% of the colonies survived at this concentration.

*Response to* cis-*platinum* Patterns of response of ovarian tumour samples to *cis*-platinum are shown in Figure 1. The response of material from untreated patients is shown in Figure 1a and b and that from pre-treated patients in Figure 1c and d. Of the 66 samples with no previous drug exposure, 39 (~60%) showed sensitivity, 16 were intermediate and 11 were resistant. However, according to the classification of Salmon *et al.* (1980), for *in vitro*/*in vivo* correlation the intermediate samples are classed as sensitive. Therefore 55/66 samples (83%) would be classified as having a response to this drug *in vitro*. This represented 41 patients (79% of total). Only 25 patients (48%), however, were truly sensitive.

Of the 5 samples exposed to *cis*-platinum previously (Figure 1c and d) one retained sensitivity while the others were markedly resistant.

*Response to adriamycin* The results with adriamycin (Figure 2) were similar to those observed for *cis*-platinum. Of the 30 samples not previously exposed to this drug (Figure 2a and b), 26 (87%) representing 76% of patients were markedly sensitive and 4 were resistant.

Of the 5 samples previously exposed to adriamycin (Figure 2c and d) only one was sensitive while the remainder were resistant.

*Response to phosphoramide mustard* The response of the 9 samples tested is shown in Figure 3. None had previously been exposed to cyclophosphamide. Two samples were sensitive and the remainder were resistant.

*Relationship between drug sensitivities*

*Untreated material* In samples from patients who had not received any cytotoxic therapy, those sensitive to adriamycin were also sensitive to *cis*-platinum with 5 exceptions. In 4 of these instances, the samples were judged to have an intermediate

**Figure 1** *In vitro* response of ovarian tumour material to *cis*-platinum; (a) and (b) from untreated patients and (c) and (d) from pre-treated patients. Sensitivity is demonstrated in (a) and (c) and resistance in (b) and (d).

**Figure 2** *In vitro* response of ovarian tumour material to adriamycin; (a) and (b) from untreated patients and (c) and (d) from pre-treated patients. Sensitivity is demonstrated in (a) and (c) and resistance in (b) and (d).

**Figure 3** *In vitro* response of ovarian tumour material to phosphoramide mustard. All patients were untreated and (a) demonstrates sensitivity and (b) demonstrates resistance.

response to *cis*-platinum. Therefore, 81% of samples with response to adriamycin were also sensitive to *cis*-platinum. This was true for both ascitic fluid and solid ovarian tumour.

Where resistance to adriamycin was observed, *cis*-platinum response was either overtly resistant or was judged to be intermediate. In no instance were resistance to adriamycin and sensitivity to *cis*-platinum observed in the same patient.

The two samples sensitive to phosphoramide mustard were sensitive to both *cis*-platinum and adriamycin. However, those samples resistant to phosphoramide mustard were also sensitive to *cis*-platinum and adriamycin with the exception of one. This sample was resistant to all 3 drugs. No relationship was observed in this small group of samples which would indicate that sensitivity *in vitro* to one alkylating agent was related to response to another.

*Pretreated material* For both *cis*-platinum and adriamycin, the 5 pretreated samples are representative of 5 patients. Four of these were resistant to both drugs and one remained sensitive after exposure. Since all patients had been exposed to both drugs, no conclusions can be drawn about the development of cross resistance.

*Clinical correlations*

Clinical follow-up revealed that some patients died without treatment and others had radiotherapy alone, while half of those who received chemotherapy were either considered unsuitable for placement in a clinical trial or the drugs were abandoned due to toxicity. Thus, of the 57 patients represented by the 71 samples available for drug study, only 48 received chemotherapy, of whom 23 received the drugs of test.

**Table II** Relationship between *in vitro* chemosensitivity and clinical outcome in patients receiving drugs of test.

| *Sample no.* | *Response to* cis-*platinum* | *Response to adriamycin* | *Chemotherapy* | *Outcome* | *Correlation* |
|---|---|---|---|---|---|
| 84 | S | — | CAP × 7 | PR | Y |
| 105 | S | — | CAP × 7 | PR | Y |
| 106 | S | — | CAP × 2 | PR | Y |
| 113 | S | — | CAP × 6 | PR | Y |
| 138 | S | S | cyclo/plat | PR | Y/– |
| 165 | S | S | CAP × 4 | PR | Y |
| 172 | S | S | CAP × 7 | PR | Y |
| 176 | S | S | CAP × 6 | CR | Y |
| 186 | S | S | CAP × 6 | CR | Y |
| 199 | S | S | cyclo/adr | CR | –/Y |
| 225 | S | — | CAP × 6 | CR | Y |
| 135 | Int | — | CAP × 5 | O | ? |
| 181 | Int | S | cyclo-plat | P | N |
| 185 | Int | S | CAP × 6 | PR | Y |
| 190 | Int | S | CAP × 1 | P | N |
| 192 | Int | S | CAP × 6 | PR | Y |
| 215 | Int | R | CAP × 4 | P | ? |
| 115 | R | — | CAP × 6 | CR | N |
| 144 | R | S | cyclo/adr | PR | –/Y |
| 213 | R | — | CAP × 6 | CR | N |
| 218 | R | R | CAP × 4 | O | ? |
| 237 | R | R | CAP × 4 | O | ? |
| 241 | R | R | CAP × 3 | O | ? |

R = resistant; S = sensitive; Int = intermediate; CAP = cyclophosphamide, adriamycin and *cis*-platinum; PR = partial response; CR = complete response; P = progression; O = stable; Y = yes; N = no; ? = equivocal.

Table II illustrates the outcome in those patients who received either *cis*-platinum or adriamycin as part of their chemotherapy. Eleven patients were judged sensitive to *cis*-platinum and 10 received it in some form. Seven achieved good partial responses and 3 obtained complete responses on the therapy as shown. Adriamycin sensitivity was known for 6 of these patients, of whom 5 received it and had a good response. For this group of patients, therefore, *in vitro* sensitivity to *cis*-platinum and adriamycin was related to a good clinical response to these drugs when received in therapy. Two of the patients who had complete response to the CAP regime were known to be sensitive to phosphoramide mustard *in vitro*, but no results were available for the remaining samples. One further patient, judged resistant to phosphoramide mustard and sensitive to *cis*-platinum and adriamycin, received cyclophosphamide as a single agent and failed to respond, but later had a good response to the CAP regime.

Of the 6 patients judged to have an intermediate response to *cis*-platinum, 4 were known to be sensitive to adriamycin of whom 3 received the CAP regime. Results here are equivocal, as 2 patients had good partial responses to the full regime but 1 progressed after only 1 pulse, although all had Stage III disease at laparotomy. If an intermediate response is classed as sensitive for the purpose of correlation (Salmon *et al.*, 1980), then patients 189 and 192 also demonstrate some relationship.

Of the 6 patients judged to be resistant to *cis*-platinum, 5 received this drug. Three were also resistant to adriamycin and are stable at this time on their CAP chemotherapy having shown no objective improvement clinically. Two further patients had complete responses to the CAP regime. Correlation in this small group of patients is poor for 2 patients and cannot yet be made for the others.

If the results are assessed on the basis of adriamycin response, there is a strong *in vitro*/*in vivo* correlation. Eight of 9 cases showed this relationship. Where adriamycin resistance occurs *in vitro*, no correlation can yet be made.

Overall, *cis*-platinum sensitivity *in vitro* has been related to good clinical response to a regime which contains it. Although it is noteworthy that sensitivity to *cis*-platinum and adriamycin have been found consistently together, it remains to be determined whether response to each element of a combination when tested singly is necessary for the combination itself to be clinically effective.

*Prediction of chemosensitivity* The relationship between clinical response to other drugs and *in vitro* response to *cis*-platinum and adriamycin in those patients who received alternative therapy is shown in Table III.

Of the 20 patients who fell into this category and have been assessed, 2 had been extensively pretreated, were resistant *in vitro* to *cis*-platinum which they had not received and died having failed to respond to further therapy (patients 177′ and 212′). However, patient 84′ had been pretreated with *cis*-platinum to which she had at first responded but died having failed to respond to another second line alkylating agent.

When *in vitro* resistance to *cis*-platinum in untreated patients is related to clinical outcome when alternative alkylating agents are received, the correlation holds good in that all 5 cases progressed. Four of these received single alkylating agents. If sensitivity to *cis*-platinum *in vitro* is related to clinical outcome, no correlation is found. Eleven of 12 such patients failed on single alkylating agent chemotherapy. Adriamycin response *in vitro* is similarly unrelated to a generalized chemosensitivity. Six of 7 patients sensitive to adriamycin progressed on their chemotherapy.

### Discussion

In this study we have shown that ovarian tumour material can be cultured successfully by clonogenic assay and that such cultures can be used to obtain *in vitro* sensitivity measurements to drugs in current use.

No relationship has been observed between the plating efficiencies recorded and either tumour type or stage of disease. Although samples of similar pathology exhibited broadly similar characteristics in culture and are clearly derived from tumour cells in the original suspensions, the growth rate of each sample is distinct and only in few instances have such rates been correlated with suggested rate of spread of disease.

Successful culture was achieved with all types of malignant material tested and tumour cell viabilities after mechanical disaggregation (≥80% for 60% of samples) were good. With the exception of peritoneal washings, culture success rates were high (69%–88%) and the total percentage successful

**Table III** Relationship between *in vitro* chemosensitivity and clinical outcome in patients receiving alternative chemotherapy.

| *Sample no.* | *Response to* cis-*platinum* | *Response to adriamycin* | *Chemotherapy* | *Outcome* | *Correlation* |
|---|---|---|---|---|---|
| 81 | S | — | Chlorambucil | P | N |
| 84′ | R | — | Treosulphan | P | Y |
| 89 | S | — | Treosulphan | O | ? |
| 90 | R | — | Cyclo Adr 5FU | P | Y |
| 101 | S | — | Cyclophosphamide | P | N |
| 102 | S | — | Treosulphan | P | N |
| 103 | S | — | Cyclophosphamide | P | N |
| 104 | R | — | Treosulphan | P | Y |
| 112 | R | — | Chlorambucil | P | Y |
| 119 | R | — | Cyclophosphamide | P | Y |
| 132 | S | — | Chlorambucil | P | N |
| 146 | S | S | Treosulphan{MTX 5FU | P | N |
| 148 | S | S | Chlorambucil | + | Y |
| 158 | S | S | Chlorambucil | P | N |
| 162 | S | S | Treosulphan | P | N |
| 165 | S | S | Cyclophosphamide | P | N |
| 177′ | R | R | Thiotepa | P | Y |
| 178 | R | S | Treosulphan | P | Y/N |
| 189 | S | S | Treosulphan | P | N |
| 212′ | R | — | Treosulphan | P | Y |

(′) = pretreated; R = resistant; S = sensitive; + = response; P = progression; O = stable; Y = yes; N = no; ? = equivocal.

culture rate of 69% compares favourably with those reported by other workers (Hamburger *et al.*, 1978; Ozols *et al.*, 1980*a*).

We have also demonstrated that 60% of samples representing 48% of previously untreated patients were sensitive to *cis*-platinum and that this number may be higher if "intermediate" values are regarded as sensitive. Only 17% of untreated samples were overtly resistant to *cis*-platinum. Similarly, 87% of untreated samples were markedly sensitive to adriamycin when tested and only 13% were resistant. These values for adriamycin compare with those of Ozols *et al.* (1980*b*) who used the same period of drug incubation but different parameters for sensitivity (70% reduction in colony forming cells at clinically achievable plasma levels).

Sensitivity to phosphoramide mustard in culture was observed in only 22% of samples, but it may be that the parameters for sensitivity discrimination were too stringent. Additionally, it has been suggested (Powers & Sladek, 1983) that phosphoramide mustard might not be the active metabolite in patients where cyclophosphamide is of therapeutic value. As the group of patients was small and few received this chemotherapy, it has not been possible to make adjustments based on clinical outcome.

Although the group of patients pretreated with *cis*-platinum and adriamycin was small, it has been demonstrated that 80% of samples taken from such patients are overtly resistant to these drugs. These figures for adriamycin again concur with those of Ozols *et al.* (1980*b*) who observed the same patterns of response in pretreated patients. Such a clear demonstration of overt resistance may be an important factor in the management of those patients where a partial response to chemotherapy is not achieved rapidly. One such patient (177′) failed on further alkylating agent chemotherapy.

Some interesting relationships between drug sensitivities have been observed. Eighty-one per cent of patients sensitive to adriamycin were also sensitive to *cis*-platinum, while those patients resistant to the drug failed to show a good response to *cis*-platinum. Resistance to adriamycin and sensitivity to *cis*-platinum were never observed in the same patient. No relationship exists, however, between sensitivity to these drugs and a sensitivity to phosphoramide mustard *in vitro*.

It is likely, therefore, that patients given a drug combination which includes *cis*-platinum and adriamycin will show a response rate of at least 40–50%. These figures are derived from the fact that 48% of untreated patients tested are sensitive to *cis*-platinum and that many of these are likely to be sensitive to adriamycin. De Paulo *et al.* (1975) quote just such response rates for adriamycin in advanced ovarian carcinoma. However, this is much less than is suggested by the *in vitro* results with adriamycin and the reasons for this discrepancy are not apparent. Cohen *et al.* (1983) quote response rates for *cis*-platinum plus adriamycin at 79–90%, and those for *cis*-platinum alone at 40–46%.

We have also shown quite clearly that *in vitro* sensitivity to *cis*-platinum and adriamycin was correlated with *in vivo* response, provided that only truly sensitive *cis*-platinum results were compared. Equivocal correlations were obtained when *in vitro* sensitivities to *cis*-platinum were regarded as intermediate. For the few samples which were resistant to *cis*-platinum and adriamycin *in vitro*, no clear correlations can yet be made.

A strong relationship exists, however, between *cis*-platinum resistance *in vitro* and failure to respond to other alkylating agents. This was true for both pretreated and untreated patients and, in the case of the untreated patients, was an unexpected finding. No correlation has been observed between adriamycin or *cis*-platinum response *in vitro* and sensitivity to other chemotherapy.

This is in contrast to the observations of the KSST group (1981) who found that adriamycin sensitivity was found to correlate with clinical response, even when the patient was not treated with this drug.

Any discussion of the relative merits of the clonogenic assay must include (a) analysis of its performance as a useful, reproducible laboratory procedure and (b) its possible clinical usefulness in the management of cancer patients and how this may be achieved.

The performance of this assay as a reproducible test in the laboratory has been established and the percentage of samples cultured successfully compares well with workers using other methodologies (Wilson & Neal, 1981).

Our culture success rates are superior to those of Williams *et al.* (1983) but in common with these and other workers, we have reservations about the low plating efficiencies associated with this method (Bertoncello *et al.*, 1982; Rupniak & Hill, 1980). As a consequence of this, too few samples are evaluable for drug study. Although 62% of our samples gave drug results in comparison to Von Hoff *et al.* (1981) who found that only 25% of samples were evaluable, in practice this number was much lower once changes in therapeutic regimes had been accounted for. Studies by Courtenay & Mills (1978) have indicated that it is possible to improve plating efficiencies and colony size by a combination of low oxygen and the presence of rat RBC, although there is not general agreement that the RBC are necessary (Pavelic *et al.*, 1980). It is clear that some modifications along these lines are necessary to improve performance of this test.

Similarly, small sample size is a problem which has greater implications for the production of drug results than is true for assays which depend on the establishment of monolayers, since the number of cells derived at the outset determines the number of tests which may be made (Bertoncello *et al.*, 1982). Samples from "first" laparotomies are often generous, but it is at "second-look" laparotomies or the tapping of effusions from patients who have progressed, that the provision of samples large enough for drug testing for possible second-line chemotherapy is vital. It is at this time either that samples cannot be obtained or the cell yield is too low. From a consideration of technical limitations, therefore, it is clear that, not only must culture conditions be modified to improve plating efficiency but that several drugs which the patient has not received be tested at one concentration alone to maximise the use of material.

The potential of the clonogenic assay in the clinical context could be realised by the adoption of several strict procedures. All the likely chemotherapy for a particular patient should be tested at the outset. Although most centres enter all suitable patients into clinical trials and few use single agent chemotherapy, the testing of 4 or 5 drugs should cover all possible treatment. Therefore, each sample should be split so that these drugs can be tested at single concentrations representing 10% of the peak plasma concentration. Sensitivity would be determined as a less than 50% survival at these concentrations. The majority of samples would be sensitive under these conditions, but a significant number may exhibit resistance to one or more drugs. If the resistance is to *cis*-platinum, it is unlikely that patients would respond to other alkylating agents.

Samples from patients who have progressed on their chemotherapy should only be tested against drugs which they have not received, if sample sizes are small. Such tests should be able to demonstrate any residual sensitivity.

In this way, the fullest possible information about likely response to chemotherapy would be made available for use in management. Although it is unlikely that fixed regimes would be changed as a result of these tests, this assay may have considerable benefit in the choice of drugs for patients who have relapsed.

It is clear that the usefulness of this assay in determining patient management remains to be tested. The value of such an *in vitro* system lies in predicting resistance in those groups of previously untreated patients who will fail to respond to their chemotherapy and in predicting sensitivity in patients who have progressed on their existing regimes or who for other reasons, such as toxicity or second-line chemotherapy for residual disease, require alternative drugs. No useful clinical information can be gained by testing drugs to which a patient is already demonstrably clinically resistant and such demonstrations do nothing to validate this system.

We are indebted to the following people for their help: Prof K.C. Calman for organising the means for clinical follow-up. Dr A.D.T. Govan, for helpful discussions and advice on tumour pathology. Prof M.C. Macnaughton, for initiating the study. The consultant staff in the Gynaecology departments of the following hospitals; Royal Infirmary, Western Infirmary, Victoria Infirmary, Royal Samaritan Hospital, Stobhill Hospital, Southern General Hospital, Thorn Hospital, Elderslie. The consultant staff in the Oncology departments at Gartnavel General Hospital, Royal and Western Infirmaries.

### References

ALBERTS, D.S., SALMON, S.E., CHEN, H.S.G. & 4 others. (1980*a*). *In vitro* clonogenic assay for predicting response of ovarian cancer to chemotherapy. *Lancet*, **ii**, 340.

ALBERTS, D.S., CHEN, H.S.G. & SALMON, S.E. (1980*b*). *In vitro* drug assay: pharmacologic considerations. In: *Cloning of Human Tumour Stem Cells*. (Ed. Salmon), New York: Alan R. Liss, p. 197.

BERTONCELLO, J., BRADLEY, T.R., CAMPBELL, J.J. & 6 others. (1982). Limitations of the clonal agar assay for the assessment of primary human ovarian tumour biopsies. *Br. J. Cancer*, **45**, 803.

COHEN, C.J., GOLDBERG, J.D., HOLLAND, J.F. & 6 others. (1983). Improved therapy with *cis*-platin regimens for patients with ovarian carcinoma (FIGO Stages III and IV) as measured by surgical end-staging (second-look operation). *Am. J. Obstet. Gynecol.*, **145**, 955.

COURTENAY, V.D. & MILLS, J. (1978). An *in vitro* colony assay for human tumours grown in immune-suppressed mice and treated *in vivo* with cytotoxic agents. *Br. J. Cancer*, **37**, 261.

DE PAULO, G.M., DELENA, M., DIRE, F., LUCIANI, L., VALAGUSSA, P. & BONADONNA, G. (1975). Melphalan versus adriamycin in advanced ovarian carcinoma. *Surg. Gynecol. Obstet.*, **141**, 899.

KSST (GROUP FOR SENSITIVITY TESTING OF TUMOURS). (1981). *In vitro* short term test to determine the resistance of human tumours to chemotherapy. *Cancer*, **48**, 2127.

HAMBURGER, A. & SALMON, S.E. (1977). Primary bioassay of human tumour stem cells. *Science*, **197**, 461.

HAMBURGER, A.W., SALMON, S.E., KIM, M.B. & 4 others. (1978). Direct cloning of human ovarian carcinoma cells in agar. *Cancer Res.*, **38**, 3438.

OZOLS, R.F., WILLSON, J.K.V., GROTZINGER, K.R. & YOUNG, R.C. (1980*a*). Cloning of human ovarian cancer cells in soft agar from malignant effusions and peritoneal washings. *Cancer Res.*, **40**, 2743.

OZOLS, R.F., WILLSON, J.K.V., WELTZ, M.D., GROTZINGER, K.R., MYERS, C.E. & YOUNG R.C. (1980*b*). Inhibition of human ovarian cancer colony formation by adriamycin and its major metabolites. *Cancer Res.*, **40**, 4109.

PAVELIC, Z.P., SLOCUM, H.K., RUSTUM, Y.M. & 5 others. (1980). Growth of cell colonies in soft agar from biopsies of different human solid tumours. *Cancer Res.*, **40**, 4151.

POWERS, J.F. & SLADEK, N.E. (1983). Cytotoxic activity relative to 4-hydroxycyclophosphamide and phosphoramide mustard concentrations in the plasma of cyclophosphamide treated rats. *Cancer Res.*, **43**, 1101.

RUPNIAK, T. & HILL, B.T. (1980). The poor cloning ability in agar of human cells from biopsies of primary tumours. *Cell Biol. Intern. Rep.*, **4**, 479.

SALMON, S.E., ALBERTS, D.S., MEYSKENS, F.L. & 6 others. (1980). Clinical correlations of *in vitro* drug sensitivity. In: *Cloning of Human Tumour Stem Cells.* (Ed. Salmon), New York: Alan R. Liss, p. 223.

SALMON, S.E. & LIU, R. (1979). Direct "wet" staining of tumour or haematopoetic colonies in agar culture. *Br. J. Cancer*, **39**, 779.

SIMMONDS, A.P., BELFIELD, A., FERGUSON, C. & FINLAY, R.J. (1981). The clonogenic assay: Its use in predictive testing. *Boll. Ist. Sieroter, Milan*, **60**, 349.

VON HOFF, D.D., CASPER, J., BRADLEY, E., SANDBACH, J., JONES, D. & MAKUCH, R. (1981). Association between human tumour colony forming assay results and response of an individual patient's tumour to chemotherapy. *Am. J. Med.*, **70**, 1027.

WILLIAMS, T.J., LIEBER, M.M., PODRATZ, K.C. & MALKASIAN, G.D. (1983). Soft agar colony formation assay for *in vitro* testing of sensitivity to chemotherapy of gynecologic malignancies. *Am. J. Obstet. Gynecol.*, **145**, 940.

WILSON, A.P. & NEAL, F.E. (1981). *In vitro* sensitivity of human ovarian tumours to chemotherapeutic agents. *Br. J. Cancer*, **44**, 189.